# Reperfusion status and postoperative blood pressure in acute stroke patients after endovascular treatment

**DOI:** 10.3389/fneur.2023.1238653

**Published:** 2023-11-10

**Authors:** Hongye Xu, He Li, Ping Zhang, Yuan Gao, Hanchen Liu, Hongjian Shen, Weilong Hua, Lei Zhang, Zifu Li, Yongxin Zhang, Pengfei Xing, Xiaoxi Zhang, Pengfei Yang, Jianmin Liu

**Affiliations:** ^1^Neurovascular Center, Naval Medical University Changhai Hospital, Shanghai, China; ^2^No. 904 Hospital of the PLA Joint Logistics Support Force, Wuxi, China; ^3^Department of Emergency, Naval Medical Center of PLA, Naval Medical University, Shanghai, China

**Keywords:** reperfusion status, postoperative blood pressure, stroke, endovascular treatment, eTICI

## Abstract

**Background and purpose:**

An aggressive lowering of blood pressure (BP) could lead to neurological worsening, particularly of the area that has not been reperfused in acute stroke patients with large vessel occlusion (LVO). We sought to investigate the association of reperfusion status and BP course following mechanical thrombectomy (MT) with outcomes in LVO.

**Materials and methods:**

Consecutive patients with LVO treated with MT between Jan 2020 to Jun 2021 were enrolled in a retrospective cohort study. Hourly systolic BP (SBP) and diastolic BP (DBP) were recorded for 72 h following MT and maximum SBP and DBP levels were identified. The Extended Thrombolysis in Cerebral Infarction (eTICI) scale was used to assess reperfusion extent. LVO patients were stratified in 2 groups based on reperfusion status: complete reperfusion (eTICI 3) and incomplete reperfusion (eTICI 2b/c). Three-month functional independence was defined as a modified Rankin Scale score of 0–2.

**Results:**

A total of 263 acute ischemic stroke patients with LVO were retrospectively evaluated. Complete reperfusion was achieved in 210 patients (79.8%). Post-MT maximum SBP over 160 mmHg was significantly related to worse functional outcome (38.1% vs. 55.7%, *p* = 0.006), higher likelihood of in-hospital mortality and 3-month mortality (19.0% vs. 6.9%, *p* = 0.004, 27.4% vs. 14.3%, *p* = 0.012). No statistical correlation was found between reperfusion status and blood pressure level (*p* > 0.05). In patients with complete reperfusion, patients with an average BP 120-140 mmHg tends to have worse functional outcome compared with 100-120 mmHg (OR = 1.77, 95%CI: 0.97–3.23, *p* = 0.061).

**Conclusion:**

High maximum SBP levels following MT are associated with an increased likelihood of 3-month functional dependence and mortality. An average BP of 100–120 mmHg tends to have better functional independence in completely reperfused patients. The effect of intensive BP control on incomplete reperfusion still warrants further investigations.

## Introduction

According to previous studies, increased systolic blood pressure (SBP) after endovascular treatment (EVT) in patients with acute ischemic stroke (AIS) has been correlated with worse clinical outcomes and an increased risk of intracranial hemorrhage (ICH) (1–7). The most recent guidelines, however, recommended a decent blood pressure target to be <180/105 mmHg in the first 24 h after endovascular treatment (8, 9). According to the second randomized controlled enhanced control of hypertension and thrombectomy stroke study (ENCHANTED-2/MT) (10), after successful reperfusion in patients with AIS, which was considered complex due to various factors, including heterogeneity in age, cause of stroke, occlusion location, and collateral status, an intensive blood pressure (BP) control of <120 mmHg was deemed harmful (11–14). An aggressive approach to lowering the BP can lead to neurological worsening in patients with incomplete reperfusion, for instance, 2b-or 2c-grade expanded thrombolysis in cerebral ischemia (eTICI) with the area that has not been reperfused. The blood pressure target in acute stroke to reduce hemorrhage after endovascular therapy trial showed a comparable effect of intensive BP control (<130 mmHg) over the standard group (130–185 mmHg) on the clinical outcome or radiological intraparenchymal hemorrhage (15). The transient focal disruption of cerebral autoregulation renders perfusion of the ischemic tissue directly dependent on systemic BP (16). Further investigation is required to determine if the harmful effect of intensive BP control is universally similar in patients with different reperfusion statuses after EVT. We investigated the association between post-mechanical thrombectomy (MT) BP level and reperfusion status in patients with stroke to explore any harmful, comparable, or beneficial effects of the intensive BP control.

## Methods

### Participants and procedure protocol

**Figure 1:**
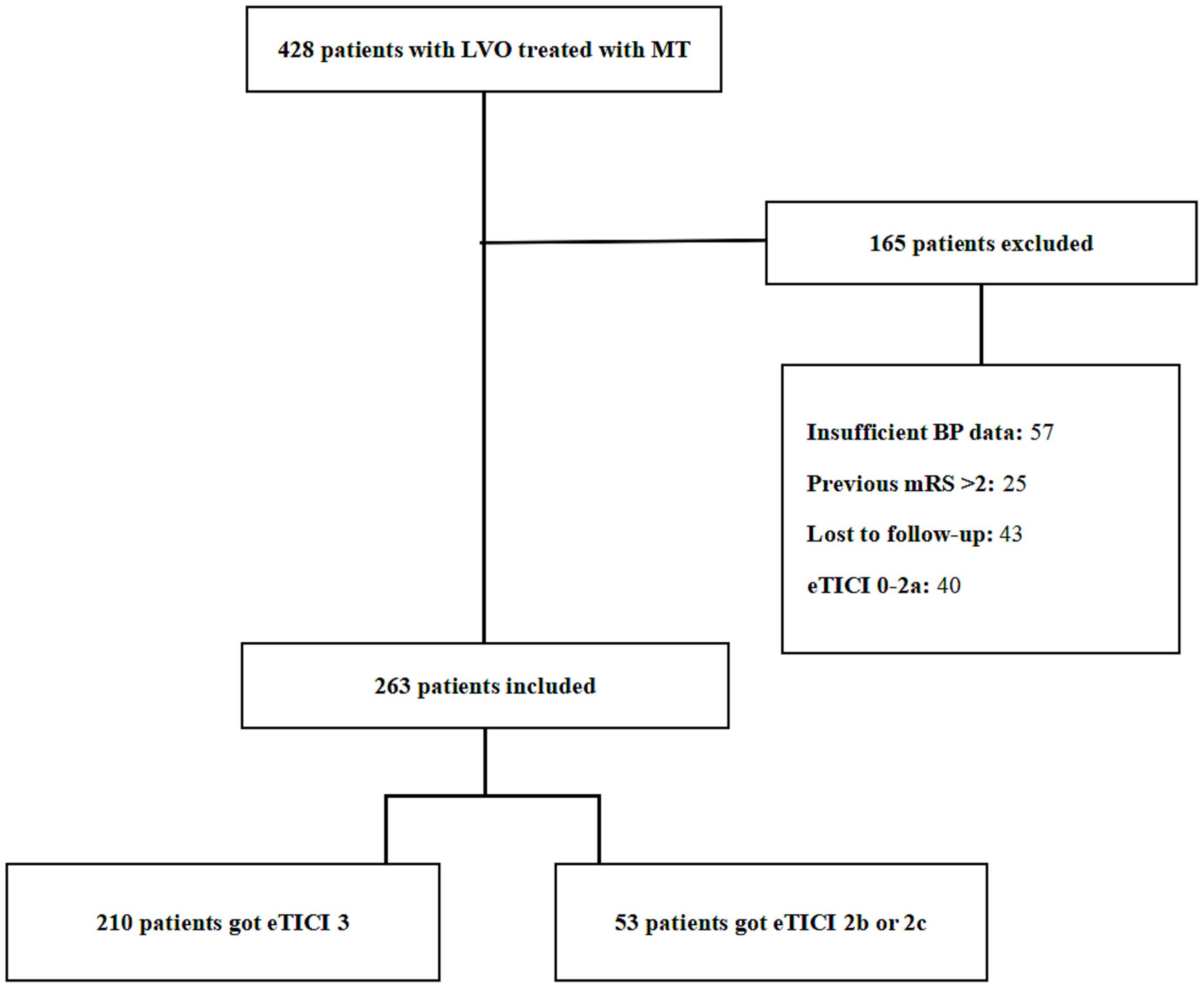
The detailed flowchart of the patient selection process. LVO, large vessel occlusion; MT, mechanical thrombectomy; BP, blood pressure; eTICI, extended Thrombolysis in Cerebral Infarction; mRS, modified Rankin scale.

From January 2020 to June 2021, we enrolled consecutive patients who presented AIS within 24 h of large vessel occlusion (LVO) and underwent MT in a comprehensive stroke center. The exclusion criteria for patients were as follows: patients who had unsuccessful reperfusion status, eTICI = 0–2a; those with insufficient BP data; patients with unfavorable previous functional status, previously modified Rankin scale (mRS) score of >2; and patients who were unable to be followed-up. The process of inclusion and exclusion is shown in Figure 1.

For evaluating the reperfusion status, eTICI (17) was used, and patients meeting the criterion were divided into the following two groups: complete reperfusion group (eTICI = 3) and incomplete reperfusion group (eTICI = 2b or 2c).

This study was approved by the Ethics Committee Boards, and the requirement for written consent was waived. All patients received verbal and written information on the collection of observational data regarding this clinical study and were free to withdraw.

### Primary and secondary outcomes

Preprocedural variable data of patients were collected from a prospectively defined clinical registry, including age, sex, comorbidities, including hypertension, diabetes mellitus, and atrial fibrillation, baseline National Institutes of Health Stroke Scale (NIHSS), admission NIHSS, admission Alberta Stroke Program Early Computed Tomography (CT) Score (ASPECTS), onset to reperfusion time (h), stroke cause according to the trial of ORG 10172 in acute stroke treatment (TOAST) criteria, and if the intravenous tissue-type plasminogen activator was administered. The primary clinical outcome was the 3-month functional independence (mRS = 0–2). The mRS was determined through in-person interviews in the follow-up clinics or telephonic interviews. Secondary outcomes included symptomatic ICH (sICH), asymptomatic ICH (aICH), and all-cause 90-day mortality. sICH was defined as any intraparenchymal, subarachnoid, or intraventricular hemorrhage on postprocedural CT associated with a ≥ 4-point increase in the NIHSS score according to the “European cooperative acute stroke study” criteria. Early neurological deterioration (END) was defined as a ≥ 4-point increase in the NIHSS score at 24 h compared with the baseline NIHSS.

### BP monitor and management protocol

Patients were admitted to the neurointensive care unit (NICU) after EVT. Their BP was monitored every 15 min before the target was achieved, and then every 1 h, with a BP cuff for 72 h. After discharge from the NICU, the BP of patients was monitored four times a day. A standardized BP measurement protocol was enacted. The BP level was decided according to the preference of the physician, with a preponderant target of <120 mmHg, 130 mmHg, or 140 mmHg, and was maintained for at least 72 h. However, because this was a real-world analysis, while not a per-protocol analysis, namely, the BP level was not precisely equal to the BP target. Intravenous BP lowering protocols guided the titration of the locally available drugs, including urapidil, nicardipine, and nitroglycerin, through repeated bolus or infusions to achieve the BP target. An SBP of <90 mmHg was set as the threshold for anti-hypertensive medication cessation and the use of intravenous fluids and inotropes, as required.

### Statistical analysis

Continuous variables are presented as mean ± standard deviation (normal distribution) and as median with interquartile range (IQR) (skewed distribution). Categorical variables are presented as percentages with their corresponding 95% confidence interval (CI). Statistical comparisons for the categorical variables between the two groups were performed using the χ^2^ test, and that for the continuous variables was performed using the unpaired *t-*test. Furthermore, the Mann–Whitney U test, one-way analysis of variance, or Kruskal–Wallis test were performed per the requirement. Univariate and multivariable logistic regression analyses were used to evaluate the association between BP level at 27 h with the clinical outcomes, including END, sICH, aICH, in-hospital mortality, 3-month mortality, and 3-month functional independence after adjusting for potential confounders (including age, sex, onset-to-reperfusion time, occlusion location, and TOAST type). The associations are presented as odds ratios (ORs) with corresponding 95% CI. Statistical significance was accepted at the *p*-value of <0.05 in the multivariable logistic regression analysis. The Statistical Package for Social Science, version 22.0 for Windows (SPSS Inc., Chicago, IL) was used for statistical analyses.

## Results

During the study period, 428 patients with LVO were treated with MT (Figure 1). The enrolled study population consisted of 263 patients (mean age = 68 ± 11 years, 63.1% men, median NIHSS score = 16 points [IQR = 9–22], and median ASPECTS score = 9 points [IQR = 7–10]) who were treated with MT and achieved reperfusion (eTICI ≥2b). Complete reperfusion (eTICI = 3) was achieved in 210 patients (79.8%) and incomplete reperfusion (eTICI = 2b or 2c) in 53 patients (20.2%). The baseline characteristics of the study population are presented in Table 1. In the complete reperfusion group, 64 (30.5%) patients had atrial fibrillation, 50 (23.8%) patients were treated for intravenous thrombolysis, and 101 (48.1%) patients were due to arteriosclerotic disease. Furthermore, 65 (31.0%) patients had internal carotid artery (ICA) occlusion, 105 (48.1%) patients had middle cerebral artery or anterior cerebral artery occlusion, 16 (7.6%) patients had vertebral artery occlusion, and 24 (11.4%) patients had basilar artery occlusion. In the incomplete reperfusion group, 25 (47.2%) patients had atrial fibrillation, 9 (17.0%) patients were treated for intravenous thrombolysis, and 21 (39.6%) patients were due to arteriosclerotic disease. Furthermore, 26 (49.1%) patients had ICA occlusion, 23 (43.4%) patients had middle cerebral artery or anterior cerebral artery occlusion, and 4 (7.5%) patients had vertebral artery occlusion. The proportion of patients with cardioembolic stroke was significantly higher in the incomplete reperfusion group than in the complete reperfusion group (*p* = 0.011), which was similar to the proportion of patients with ICA occlusion (49.1% and 31.0% in the incomplete and complete reperfusion group, respectively). The risk of sICH in the complete reperfusion group (5.8%) was similar, without any statistically significant difference, to that in the incomplete reperfusion group (5.7%) (*p* = 1.000). Similarly, END occurred in 13.8 and 18.9% of patients in the two groups, with no statistical significance (*p* = 0.586). However, the risk of aICH was significantly higher in the incomplete reperfusion group (15.5%) than in the complete reperfusion group (28.3%) (*p* = 0.031). The proportion of patients with 3-month functional independence tend to be higher in the complete reperfusion group (50.5%) than in the incomplete reperfusion group (36.7%). Nevertheless, the proportion of patients with 3-month mortality tend to be lower in the complete reperfusion group (19.1%) than in the incomplete reperfusion group (24.5%).

**Table 1:** Baseline characteristics of the study population dichotomized by reperfusion status^*^.

Variables	Complete reperfusion (*n* = 210)	Incomplete reperfusion (*n* = 53)	*p* value
Age, *y*, mean ± SD	67.7 ± 11.0	70.7 ± 11.0	0.085
Male, *n* (%)	135 (64.3)	31 (58.5)	0.435
Hypertension, *n* (%)	173 (82.4)	40 (75.5)	0.880
Diabetes mellitus, *n* (%)	112 (53.3)	26 (49.1)	0.577
Atrial fibrillation, *n* (%)	64 (30.5)	25 (47.2)	0.022
Baseline NIHSS score, points, median (IQR)	15 (9, 21)	16 (13, 22)	0.389
Baseline ASPECTS, points, median (IQR)	9 (7, 10)	8 (5, 9)	0.021
IV thrombolysis treated, *n* (%)	50 (23.8)	9 (17.0)	0.287
TOAST type			0.011
Cardioembolism	60 (28.6)	26 (49.1)	
Arteriosclerosis	101 (48.1)	21 (39.6)	
Others	49 (23.3)	6 (11.3)	
Occlusion site, *n* (%)			0.003
ICA	65 (31.0)	26 (49.1)	
MCA/ACA	105 (50.0)	23 (43.4)	
VA	16 (7.6)	4 (7.5)	
BA	24 (11.4)	0 (0.0)	
Tandem lesion, *n* (%)	37 (17.7)	10 (19.2)	0.798
Admission SBP, mmHg, mean (SD)	153.8 ± 28.3	147.2 ± 30.9	0.202
Admission DBP, mmHg, mean (SD)	86.1 ± 17.2	84.5 ± 18.2	0.602
72 h SBP_mean_, mmHg, mean (SD)	120.8 ± 15.0	117.6 ± 21.2	0.216
72 h DBP_mean_, mmHg, mean (SD)	68.0 ± 10.7	66.0 ± 13.3	0.247
Onset to reperfusion time, hour, mean (SD)	10.0 ± 9.1	10.2 ± 8.8	0.856
Symptomatic intracranial hemorrhage, *n* (%)	12 (5.8)	3 (5.7)	1.000
Asymptomatic intracranial hemorrhage, *n* (%)	32 (15.5)	15 (28.3)	**0.031**
Early neurological deterioration, *n* (%)	29 (13.8)	10 (18.9)	0.388
3-month functional independence, *n* (%)	103 (50.5)	18 (36.7)	0.083
3-month mortality, *n* (%)	39 (19.1)	12 (24.5)	0.400

*p value in bold indicates statistical significance; complete reperfusion defined as an expanded Thrombolysis in Cerebral Infarction [eTICI] score of 3 after MT; Incomplete reperfusion defined as an expanded Thrombolysis in Cerebral Infarction [eTICI] score of 2b or 2c after MT; IQR interquartile range; SD, standard deviation; ICA, internal carotid artery; MCA, middle cerebral artery; ACA, anterior cerebral artery; VA, vertebral artery; BA, basilar artery; SBP, systolic pressure; DBP, diastolic blood pressure.

The comparison of the baseline characteristics and outcomes in the two groups of post-MT maximum SBP among patients with successful reperfusion after EVT is presented in Table 2. Although the baseline characteristics of the two groups did not differ, their clinical outcomes were notably different. Both groups documented similar SBP and diastolic BP (DBP) at admission (SBP: 150.6 ± 28.9 mmHg versus 154.0 ± 28.9 mmHg, *p* = 0.381; DBP: 86.7 ± 18.3 mmHg versus 85.1 ± 16.7 mmHg, *p* = 0.489). In patients with a maximum SBP of >160 mmHg, the risk of sICH significantly increased (9.7% versus 2.7%, *p* = 0.029). The risk of aICH was similar (17.7% versus 18.5%, *p* = 1.000). END observation in both groups was comparable in patients with a maximum SBP of >160 mmHg (18.1% versus 12.2%, *p* = 0.222). However, in-hospital and 3-month mortality rates were significantly higher (19.0% versus 6.9%, *p* = 0.004, 27.4% versus 14.3%, *p* = 0.012, respectively). The 3-month functional independence was significantly higher in patients with a maximum SBP of <160 mmHg (38.1% versus 55.7%, *p* = 0.006).

**Table 2:** Baseline characteristics and outcomes of the study population dichotomized by maximum SBP within 72 h post MT*.

Variables	Maximum SBP > 160 mmHg (*n* = 121)	Maximum SBP < 160 mmHg (*n* = 139)	*p* value
Age, y, mean ± SD	69.8 ± 10.8	67.1 ± 11.2	0.058
Male, *n* (%)	70 (60.3)	96 (65.3)	0.441
Hypertension, *n* (%)	100 (82.6)	113 (81.3)	0.872
Diabetes mellitus, *n* (%)	69 (57.0)	69 (49.6)	0.263
Atrial fibrillation, *n* (%)	43 (35.5)	46 (33.1)	0.696
Baseline NIHSS score, points, median (IQR)	16 (8, 20)	16 (10, 23)	0.099
Baseline ASPECTS, points, median (IQR)	9 (7, 10)	9 (7, 10)	0.723
IV thrombolysis treated, *n* (%)	21 (18.1)	38 (25.9)	0.140
TOAST type			0.402
Cardioembolism	36 (29.7)	51 (36.7)	
Arteriosclerosis	59 (48.8)	57(41.0)	
Others	26 (21.5)	31 (22.3)	
Occlusion site, *n* (%)			0.105
ICA	49 (40.5)	49 (35.3)	
MCA/ACA	47 (38.8)	73 (52.5)	
VA	10(8.3)	8 (5.8)	
BA	15 (12.4)	9 (0.6.4)	
Tandem lesion, *n* (%)	20 (17.2)	27 (18.6)	0.872
Admission SBP, mmHg, mean (SD)	150.6 ± 28.9	154.0 ± 28.9	0.381
Admission DBP, mmHg, mean (SD)	86.7 ± 18.3	85.1 ± 16.7	0.489
Onset to reperfusion time, hour, mean (SD)	10.1 ± 6.7	10.0 ± 10.6	0.925
Complete reperfusion, *n* (%)	92 (79.3)	118 (80.3)	0.878
Symptomatic intracranial hemorrhage, *n* (%)	11 (9.7)	4 (2.7)	**0.029**
Asymptomatic intracranial hemorrhage, *n* (%)	20 (17.7)	27 (18.5)	1.000
Early neurological deterioration, *n* (%)	21 (18.1)	18 (12.2)	0.222
In-hospital mortality, *n* (%)	22 (19.0)	10 (6.9)	**0.004**
3-month functional independence, *n* (%)	43 (38.1)	78 (55.7)	**0.006**
3-month mortality, *n* (%)	31 (27.4)	20 (14.3)	**0.012**

*p value in bold indicates statistical significance. SD, standard deviation; IQR, interquartile range; ICA, internal carotid artery; MCA, middle cerebral artery; ACA, anterior cerebral artery; VA, vertebral artery; BA, basilar artery; SBP, systolic pressure; DBP, diastolic blood pressure.

The multivariable analysis and associations of different post-MT SBP averages with 3-month functional dependence after adjustment for potential confounders in the subgroup of patients with LVO with complete and incomplete reperfusion following MT are presented in Table 3. Interestingly, no association was found between postprocedural average SBP levels and risk of 3-month functional dependence, END, sICH, in-hospital mortality, and 3-month mortality (*p* > 0.05) (Table 3; Supplementary Table S2). Moreover, patients with an average SBP of 100–120 mmHg tended to have the best 3-month functional outcome in the complete reperfusion group, and those in the incomplete reperfusion group had worse outcomes; however, the difference was statistically insignificant. In the complete reperfusion group, the OR of the 3-month functional outcome of patients with 120–140 mmHg over those with 100–120 mmHg was 1.77 (95% CI, 0.97–3.23, *p* = 0.061). Additionally, no linear relationship was found between post-MT SBP and clinical outcomes based on the reperfusion status (*p* > 0.05) (Supplementary Table S1).

**Table 3:** Multivariate analysis in the overall patients*.

	Complete reperfusion		Incomplete reperfusion	
	Outcome OR (95%CI)	*p value*	Outcome OR (95%CI)	*p value*
3-month functional independence				
<100 mmHg	1.23 (0.38–4.11)	0.705	0.42 (0.05–3.83)	0.574
100-120 mmHg	1.00 (reference category)	NA	1.00 (reference category)	NA
120-140 mmHg	1.77 (0.97–3.23)	0.061	0.74 (0.20–2.79)	0.657
>140 mmHg	2.57 (0.69–9.54)	0.197	0.29 (0.14–0.61)	0.123
3-month mortality				
<100 mmHg	0.43 (0.05–3.63)	0.681	3.25 (0.34–31.07)	0.544
100–120 mmHg	1.00 (reference category)	NA	1.00 (reference category)	NA
120-140 mmHg	1.51 (0.70–3.26)	0.289	0.81 (0.18–3.60)	1.000
>140 mmHg	1.15 (0.22–5.99)	1.000	0.77 (0.59–1.00)	1.000

*Association of achieved average systolic blood pressure during the first 72 h following mechanical thrombectomy with clinical outcomes on multivariable logistic regression models adjusting for age, sex, occlusion location, toast type and onset to revascularization time.

## Discussion

This study indicated that a maximum SBP level of >160 mmHg during the first post-MT 72 h is related to a higher likelihood of worse clinical outcomes, including in-hospital mortality, END, 3-month mortality, and 3-month functional dependence. Multivariate regression analysis did not reveal any relationship between the reperfusion status and BP level on the clinical outcomes. Additionally, patients with complete reperfusion tended to have the best functional outcome with an average SBP of 100–120 mmHg. The main finding of this study is that the association between the post-MT BP target and clinical outcome is comparable with the previous studies (18–21).

ENCHANTED-2/MT is a multicenter, open-label, blinded-endpoint, randomized controlled trial that compares the safety and efficacy of more intensive BP lowering treatment (<120 mmHg) with less intensive treatment targets (140–180 mmHg) in patients with increased BP after reperfusion with EVT (10). The likelihood of poor functional outcome was greater in the more intensive treatment group (cOR = 1.37 [95% CI: 1.07–1.76]) and increased numbers of END (cOR = 1.53 [95% CI, 1.18–1.97]) and major disability at 90 d (OR = 2.07 [95% CI, 1.47–2.93]), but no significant differences in symptomatic intracerebral hemorrhage were observed (10). Furthermore, the study found worse outcomes in the intensive group similar to that of incomplete reperfusion, for instance, eTICI 2b, an aggressive approach to lowering the BP could lead to neurological worsening, particularly in the incompletely reperfused area. We hypothesized that the reperfusion status was related to the microcirculation of the local brain tissue, and the good microcirculation may tolerate the lower blood pressure. We will further carry out relevant animal experiments to prove our conjecture.

The AURORA meta-analysis of thrombectomy for anterior circulation stroke >6 h after the last known well revealed that the post-MT functional independence was 45.9%, with a mortality rate of 16.5% and an sICH rate of 5.3% (22). Herein, the rates of functional independence, mortality, and sICH of patients with complete and incomplete reperfusion were 50.5%, 19.1% and 5.8%, 36.7%, and 24.5% and 5.7%, respectively, which were comparative to the results of the abovementioned study. Attempts to achieve an eTICI score of 3 with complete reperfusion might be the priority in performing EVT; however, considering that >half of the patients still had a residual disability at 3 months despite successful reperfusion, it is important to consider potential factors, including BP, anesthesia, blood glucose, and body temperature, that might help improve the functional outcomes. ENCHANTED-2/MT was the first randomized controlled trial that indicated that a more-intensive BP control might be harmful compared with that of <120 mmHg in patients with LVO that achieved successful reperfusion (10). An alternative interpretation is that the patients with residual unreperfused areas might have deteriorated outcomes after intensive BP control. More recently, the OPTIMAL-BP trial was terminated earlier due to safety concern and found intensive BP management (<140 mmHg) for 24 h led to a lower likelihood of functional independence at 3 months compared with conventional BP management (140–180 mmHg) (23). Interestingly, we did not find any significant relationship between post-MT BP and the reperfusion status, suggesting that further investigations were warranted for more conclusive information.

In a study, considerable discrepancy regarding BP target was reported among different institutions and physicians (24). An online survey of neurointerventionalists, neurointensivisits, and neurologists from different comprehensive stroke centers revealed that the BP target was determined by a team of physicians in a collaborative fashion (*n* = 30, 52%) and individualized on a case-by-case basis (*n* = 39, 67%) in most institutions. Among them, 36% of physicians preferred an SBP target in the 120–139 mmHg range and 5% preferred that of <120 mmHg. In another online survey endorsed by the European Society of Intensive Care Medicine (24), 54% of participants chose a BP target of <160/90 mmHg. Most participants believed that an eTICI 2b/c versus eTICI 3 reperfusion status would not affect the optimal management of post-MT SBP.

There are some limitations of this study. Because of the retrospective nature of this study, it was subjected to the biases inherent to this type of analysis. The BP levels were decided according to the preference of the physician, and not based on randomization. Furthermore, we could not evaluate the hypothesis that if BP target affected clinical prognosis, considering the information regarding the type of BP target was not adequate in the study cohort. Moreover, we did not measure the final infarct volume; therefore, we could not evaluate its association with BP level. Instead, END, which partially indicates the deterioration of cerebral infarction, was evaluated. There is also a higher proportion of patients with cardioembolic stroke in the incomplete reperfusion group, which may lead to bias. According to previous immunohistochemical staining study, platelet-rich thrombi were associated with a smaller prevalence of TICI 3 compared to platelet-poor thrombi, which is in coincidence with our result (25). Finally, the observational study design did not allow us to establish a cause–effect relationship between post-MT BP levels and functional independence in patients with LVO.

## Conclusion

Our study showed the preliminary data indicating that SBP of >160 mmHg, following MT, was associated with an increased likelihood of 3-month mortality and functional dependence in patients with LVO. An average SBP between 100–120 mmHg tended to have a better 3-month functional independence in patients with complete reperfusion, whereas patients with incomplete reperfusion showed worse 3-month functional independence. Further investigation is needed to determine if an incomplete reperfused area can affect the BP target.

## Data availability statement

The raw data supporting the conclusions of this article will be made available by the authors, without undue reservation.

## Ethics statement

Ethical review and approval was not required for the study on human participants in accordance with the local legislation and institutional requirements. Written informed consent from the patients/participants or patients/participants legal guardian/next of kin was not required to participate in this study in accordance with the national legislation and the institutional requirements.

## Author contributions

HX, HL and PZ: drafted this manuscript. YG, HaL, HS, WH, LZ, ZL, YZ, PX, and JL: contributed in statistical analysis, data collection, and imaging analysis. XZ and PY: designed this study. All authors contributed to the article and approved the submitted version.

## Figures and Tables

**Table:** 

aICH	asymptomatic ICH
AIS	acute ischemic stroke
ASPECTS	Alberta stroke program early CT score
BP	blood pressure
CI	confidence interval
CTA	CT angiography
DBP	diastolic blood pressure
END	early neurological deterioration
eTICI	expanded thrombolysis in cerebral infarction
IQR	interquartile range
IVT	IV thrombolysis
LVO	large vessel occlusion
MCA	middle cerebral artery
mRS	modified rankin scale
MT	mechanical thrombectomy
NIHSS	NIH stroke scale
OR	odds ratio
RCT	randomized controlled trial
SBP	systolic blood pressure
sICH	intracranial hemorrhage
