# Supplementary material for: Reperfusion status and postoperative blood pressure in acute stroke patients after endovascular treatment
Source: Front Neurol. 2023 Nov 10;14:1238653. doi: 10.3389/fneur.2023.1238653 (PMC10668020; doi:10.3389/fneur.2023.1238653)
Supplement: Supplementary file 1 [file Data_Sheet_1.pdf]

e-table 1. Linear relationship between average BP level and clinical outcome according to reperfusion status.

	Complete reperfusion			Incomplete reperfusion	
	r value	*p*		*r* value	*p*
sICH	-0.033	0.637		0.050	0.725
aICH	0.026	0.711		-0.206	0.142
END	-0.001	0.987		-0.093	0.512
In-hospital mortality	-0.012	0.867		-0.151	0.286
3-month functional independence	0.130	0.065		-0.004	0.979
3-month mortality	0.048	0.497		-0.170	0.247

e-table 2.

	Complete reperfusion			Incomplete reperfusion	
	OR (95%CI)	*p*		OR (95%CI)	*p*
sICH					
<100mmHg	1.03(0.11-9.56)	1.000		0.94(0.84-1.06)	1.000
100-120mmHg	1.00 (reference category)	NA		1.00 (reference category)	NA
120-140mmHg	0.64(0.18-2.31)	0.522		1.33(0.11-15.96)	1.000
>140mmHg	1.34(0.14-12.68)	0.586		0.94(0.84-1.06)	1.000
aICH					
<100mmHg	0.35(0.04-2.91)	0.450		0.18(0.02-1.86)	0.179
100-120mmHg	1.00 (reference category)	NA		1.00 (reference category)	NA
120-140mmHg	1.02(0.43-2.43)	1.000		0.21(0.05-0.83)	0.021
>140mmHg	0.65(0.08-5.64)	1.000		0.47(0.28-0.78)	0.218
END					
<100mmHg	1.77(0.42-7.48)	0.431		0.65(0.06-7.32)	0.726
100-120mmHg	1.00 (reference category)	NA		1.00 (reference category)	NA
120-140mmHg	1.42(0.69-2.93)	0.345		0.59(0.13-2.77)	0.502
>140mmHg	1.63(0.38-6.96)	0.449		0.77(0.59-1.00)	1.000
In-hospital mortality					
<100mmHg	0.56(0.07-4.77)	1.000		0.65(0.06-7.32)	1.000
100-120mmHg	1.00 (reference category)	NA		1.00 (reference category)	NA
120-140mmHg	0.89(0.36-2.24)	0.810		0.42(0.08-2.20)	0.407
>140mmHg	1.61(0.30-8.64)	0.630		0.77(0.59-1.00)	1.000
3-month functional independence					
<100mmHg	1.23(0.38-4.11)	0.705		0.42(0.05-3.83)	0.574
100-120mmHg	1.00 (reference category)	NA		1.00 (reference category)	NA
120-140mmHg	1.77(0.97-3.23)	0.061		0.74(0.20-2.79)	0.657
>140mmHg	2.57(0.69-9.54)	0.197		0.29(0.14-0.61)	0.123
3-month mortality					
<100mmHg	0.43(0.05-3.63)	0.681		3.25(0.34-31.07)	0.544
100-120mmHg	1.00 (reference category)	NA		1.00 (reference category)	NA
120-140mmHg	1.51(0.70-3.26)	0.289		0.81(0.18-3.60)	1.000

>140mmHg	1.15(0.22-5.99)	1.000		0.77(0.59-1.00)	1.000
